# The Medical Profession of the United States

**Published:** 1846-05

**Authors:** 


### THE MEDICAL PROFESSION OF THE UNITED STATES.

It must be admitted that we, the physicians of the United States, are very much dissatisfied with ourselves. On all sides we hear complaints of the state of the profession—of defects in medical schools and in primary education—of the progress of quackery, and the necessity for a higher standard in medicine. No one, we believe, has gone quite so far in depreciating his profession as Dr. J. N. Davis, the projector of the New York Medical Convention. In the Circular, of which he is the author, addressed to the medical societies and colleges of the country, he holds the following language. We find it quoted by Professor Paine in his Valedictory Lecture. It so happens that we did not read the Circular, and this must be our apology to our brethren for not vindicating them from such aspersions. The quotation runs as follows:

"There may, indeed, be found here and there, a mind, so endowed by nature, that despite of all the contracting and evil influences of such a system, will rise superior to every obstacle, and shaking off one embarrassment after another, will roam over, and revel in, all the fields of science untrammeled and free. And it is such that constitute the ornament and honor of our profession.

"But far otherwise is it with the great mass; the ninety-nine out of every hundred. With no practical knowledge of chemistry and botany; with but a smattering of anatomy and physiology, hastily caught during a sixteen weeks' attendance on the anatomical theatre of a medical college; with still less of real pathology; they enter the profession, having mastered just enough of the details of practice to give them the requisite self-assurance for commanding the confidence of the public; but without either an adequate fund of knowledge, or that degree of mental discipline, and habits of patient study, which will enable them ever to supply their defects.

Hence they plod on through life, with a fixed routine of practice, consisting of calomel, antimony, opium, and the lancet, almost as empyrically applied as is cayenne pepper, lobelia, and steam, by another class of men. And how can this ever be otherwise, while medical students, without regard to their previous qualifications, are shut up three years in the office of a country or village physician, sent out to one or two courses of lectures, and then honored with the title of M.D.? Indeed so radical are the defects in our present system of medical education, and so glaring are their effects on the condition and usefulness of the profession, that no observing man will charge us with exaggeration in the foregoing statements."

If a copy of Professor Paine's lecture has been sent to us we have been so unfortunate as not to receive it, and it was only a few days since that we had the pleasure of meeting with it on the table of a friend. From the title-page we perceive that in the short space of one month the lecture has reached the *sixth* edition. The reader will not be surprised to hear of this unusual popularity, when he is told that the lecture is a "defence of the medical profession in the United States" against these charges. Dr. Paine has long maintained that as practitioners, American physicians are the best in the world. He is sensible to defects which exist; he admits that much may be done towards correcting them by wise concert of action; but he is naturally indignant at such accusations as those of which specimens are given in the paragraphs above quoted. His defence is a manly one, and we wish it could be read by every physician in the United States. We are glad to learn that it has already attracted so much attention, and hope the interest it has awakened will not soon subside.

On one point Professor Paine is eminently successful;—he shows conclusively, that the author of the Circular is not himself a scholar. But we are not able to perceive that he helps his argument for the American profession, by proving that a member who sets up to be one of its leaders is not able to spell his own language. Such proof may be very unfortunate for the projector of the convention, but it cannot be said to reflect much credit upon the profession, "the standard" of which he professes such a solicitude to raise. In an evil hour, this gentleman addressed the Circular calling the Convention to the Faculty of the New York University, and Dr. Paine publishes it to the world as it was written. Here it is.

"The above Circular," says the Chairman, "is an Extract from the proceedings of the New York State Medical Society at its last *anual* meeting in Feb., 1845. The object of the proposed convention is sufficiently indicated in the preamble and resolutions itself. The Committee, in the discharge of their duty, have corresponded with the Medical Societies and Colleges in all the States where such institutions exist, and the replies which have been received are *unanymously* in favor of the proposed convention.

"Indeed, several Colleges have already appointed delegates; consequently, there is no longer any doubt but that a convention *will be held.* If the subject has not already been before the Faculty of the Medical Department of the University, will you not, without further delay, bring it to their *notise?*

"(Signed,) J. N. DAVIS."

The italics are Dr. Paine's; the spelling is Dr. Davis's own. The extracts need no comments. One, as he reads them, can hardly help recalling to mind the wise caution given to dwellers in "glass houses;" but, alas, how seldom is vouchsafed to us the inestimable "gift" of "seeing ourselves as others see us!"

